# *Lavandula angustifolia* and *Cyperus rotundus* Extract Treatments Ameliorate Copper Toxicity in Rats

**DOI:** 10.1002/brb3.70624

**Published:** 2025-07-07

**Authors:** Mandana Salari, Faezeh Dahoee, Seyed Jamilaldin Fatemi, Manijeh Dogani

**Affiliations:** ^1^ Department of Chemistry, Faculty of Science Shahid Bahonar University of Kerman Kerman Iran; ^2^ Department of Biology, Faculty of Science Shahid Bahonar University of Kerman Kerman Iran

**Keywords:** chelation therapy, copper toxicity, *Cyperus rotundus*, deferasirox, gene expression, lavender, learning and memory, stress oxidative

## Abstract

**Introduction:**

Prolonged exposure to copper (Cu) induces oxidative stress, which causes neuronal damage and contributes to cognitive decline.

**Methods:**

In this study, we assessed the free radical scavenging potential of *Lavandula angustifolia* and *Cyperus rotundus* extracts and compared their ability to counteract the harmful effects of copper in male rats. These plants were selected on the basis of their well‐documented antioxidant properties and traditional medicinal uses.

**Results:**

The results showed that both extracts inhibited over 80% of 2,2‐diphenyl‐1‐picrylhydrazyl (DPPH) radicals compared to control groups. Additionally, Cu accumulates in the rats’ kidneys, liver, and brain, impairing their cognitive performance and liver and kidney function. Exposure to copper also increased malondialdehyde (MDA) and H_2_O_2_ levels and the expression of TNF‐α, IL‐6, IL1‐B, and Caspase‐3 genes while decreasing that of the BDNF. However, treatment with the herbal extracts significantly reduced Cu accumulation and improved the animals’ learning and memory. It also decreased MDA and H_2_O_2_ levels and regulated the genes’ expression against Cu‐induced oxidative stress and inflammation in the animals’ brains. The effectiveness of these extracts was almost similar.

**Conclusion:**

These results highlight the potential therapeutic benefits of *L. angustifolia* and *C. rotundus* extracts in combating copper toxicity.

## Graphical abstract

High levels of copper can lead to spatial learning and memory disorders. Extracts from *L. angustifolia* and *C. rotundus* have the potential to remove copper from the body and reduce its harmful effects. They achieve this by increasing the expression of the BDNF gene while decreasing the expression of IL‐6, IL1‐B, Caspase‐3, and TNF‐α genes in the hippocampus due to their antioxidant properties.

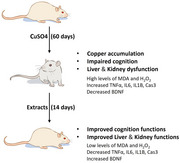

## 1 Introduction

Copper (Cu) is a trace element essential to living organisms. It functions as a co‐factor for structural and catalytic enzymes, but its high concentrations can be toxic (Pavelková et al. 2018; Pohanka 2019). According to the Agency for Toxic Substances and Disease Registry (ATSDR), the daily requirement for Cu is 1 mg, with most diets supplying 1–5 mg/day (Nagel 2021). Increased levels of free Cu^2+^ stimulate the production of hydroxyl free radicals (•HO), cause oxidative damage, and are associated with the pathogenesis of various disorders (Gözde 2012; Halliwell and Gutteridge 1990; Uriu‐Adams and Keen 2005). Excessive exposure to Cu has been shown to be associated with cellular lesions present in Wilson's disease, Alzheimer's disease (AD), and memory disorders in humans (Dorsey and Ingerman 2004; Tchounwou et al. 2008). Moreover, hepatic Cu accumulation can result in hepatocellular damage, cirrhosis, and liver failure. In the kidneys, excessive Cu can cause tubular damage, leading to renal dysfunction and potential kidney failure (Attia et al. 2021; Borobia et al. 2022).

At the molecular level, excess Cu exposure triggers a cascade of events that involve oxidative stress, inflammation, and apoptosis. Elevated Cu^2^⁺ levels enhance the production of reactive oxygen species (ROS), leading to lipid peroxidation, protein oxidation, and DNA damage. These oxidative insults activate inflammatory pathways, resulting in the release of pro‐inflammatory cytokines, such as TNF‐α, IL‐6, and IL‐1β. The sustained inflammatory state, in turn, not only exacerbates oxidative stress but also primes neuronal cells for apoptosis, as indicated by the upregulation of Caspase‐3, an essential executioner of programmed cell death.

The removal of heavy metals from the body through the administration of chelating agents has been a long‐standing treatment for metal poisoning. Nevertheless, the therapeutic use of these agents is associated with potential adverse effects and the risk of drug interactions, mandating diligent patient surveillance (Flora and Pachauri 2010; V. Singh et al. 2024). In this context, plant extracts with chelating agents offer several advantages over common chemical chelating agents. They are sourced from natural materials, reducing environmental impact, and typically have fewer side effects. Plant extracts contain a diverse range of compounds that can exhibit chelating properties, providing a broader and potentially more effective chelation profile (Adusei et al. 2019; Wong et al. 2014). Moreover, plants are rich in phenolic and flavonoid compounds with multiple biological functions, such as antioxidant activities, free radical sweeping capacities, anti‐inflammatory, and anti‐carcinogenic activities (Miller 1996; Samandari Bahraseman et al. 2022; Yousif et al. 2012). These compounds possess strong reducing properties, act as donors of electrons and hydrogen, and can effectively quench singlet oxygen (Hazra et al. 2010). Therefore, they hold promising potential to neutralize the hazardous effects of heavy metals on human bodily functions.

The selection of *Lavandula angustifolia* (generally called lavender) and *Cyperus rotundus* (commonly known as nutgrass) for this study was based on their well‐documented antioxidant properties and traditional medicinal uses. Lavender is a species of the Lamiaceae family, and its antioxidant (Spiridon et al. 2011) and antimicrobial properties (Adaszyńska‐Skwirzyńska and Dzięcioł 2017; Moon et al. 2007) have been reported already. Moreover, lavender scent inhalation can prevent stress, anxiety, and depression (Kianpour et al. 2016). Similarly, *C. rotundus* is a type of sedge found in Africa, the southern and central regions of Europe, and the southern region of Asia. This plant is known for its various health benefits, such as antimicrobial, anti‐inflammatory, wound healing, memory improvement, antidiabetic, gastroprotective, and hepatoprotective activities (Adsersen et al. 2006; Duarte et al. 2005; Kilani et al. 2008). *C. rotundus* is known for its high content of phenolic compounds and flavonoids, which contribute to its strong free radical scavenging activity. Similarly, *L. angustifolia* has been widely studied for its potent antioxidant and neuroprotective effects. These attributes make both plants promising candidates for mitigating oxidative stress and cognitive dysfunction caused by Cu toxicity.

The aim of this study was to assess the antioxidant activity of extracts from *L. angustifolia* and *C. rotundus* and evaluate their potential to mitigate memory and learning dysfunction caused by Cu. Toward this end, we examined the effect of *C. rotundus* and *L. angustifolia* extract treatments on the performance of animals that had been subjected to CuSO_4_ for 2 months. Furthermore, we evaluated the serum levels of animals’ liver and kidney factors. Moreover, we assessed the malondialdehyde (MDA) and H_2_O_2_ levels and the expression of BDNF, TNF‐α, IL‐6, IL1‐B, and Caspase‐3 genes in the animals’ brains. The Cu content accumulated in the animals’ tissues was also measured before and after the treatment period.

## 2 Materials and Methods

### 2.1 Materials

The 2,2‐diphenyl‐1‐picrylhydrazyl (DPPH) chemical compound was acquired from Sigma‐Aldrich, based in St. Louis, MO, USA. Copper(II) sulfate pentahydrate (CuSO_4_·5H_2_O), ethanol, and methanol were purchased from Merck Chemicals Co. (Germany).

### 2.2 Herbal Extract Preparation

*L. angustifolia* and *C. rotundus* samples were procured from local markets of the Neyshabur (Khorasan Razavi province) and Shiraz (Fars province) in Iran, respectively. The herb samples were transferred to the laboratory and were authenticated by botanical expert Dr. Mansour Mirtadzadini. The authentication process involved a thorough examination of the plant morphology and comparison with herbarium specimens. The samples were then washed with distilled water, air‐dried in the shade, and powdered. After that, the extraction procedures were completed by immersing 100 g of each *L. angustifolia* and *C. rotundus* extract powder in aqueous methanol (80% v:v) for 72 h at lab temperature in the darkroom, according to the maceration method (Di Donato et al. 2018). Afterward, an ultrasonic bath (35°C, 15 min) was used to complete the extraction process. The samples were then filtered, and the methanol was evaporated via a rotary evaporator at 40°C. The process concluded with the drying of the herbal extracts at room temperature, after which they were stored at 4°C until further use.

### 2.3 Assessment of the Extracts’ Radical Scavenging Activity

The potential of the extracts on the inhibiting of DPPH radicals was evaluated according to Khorrami et al. (2019) with slight modification. Briefly, 100 µL of diverse concentrations of each extract (10, 20, 40, 60, 80, and 100 µg/mL) were added to 150 µL of DPPH dissolved in methanol (0.25 mM) in a 96‐well plate. The control was the mixture of deionized water and the DPPH solution. The plate was then shaken for a short time and allowed to stand for 30 min in the dark. Next, a microplate reader (BioTek ELX 800 ELIZA Reader) was used to measure the absorbance of each well at 517 nm. The percentage of DPPH radical inhibition was calculated according to the following equation:

(1) $$\mathrm{Inhibition}\;\left(\%\right)=\left[\left(A_{0}-A_{1}\right)/A_{0}\right]\times 100$$

where *A*_0_ represents the absorbance of the control, whereas A_1_ represents the absorbance of DPPH exposed to the extract.

Linear regression analysis was performed to identify the concentration of each extract that is required to reduce 50% of DPPH radicals, which is known as IC_50_.

### 2.4 Animals

For this study, we used 42 male Wistar rats with a weight range of 200–250 g. The animals were obtained from the Laboratory Animal Maintenance and Breeding Center of Kerman University of Medical Sciences. They were kept in animal houses of Shahid Bahonar University of Kerman, Iran, and provided with freely available water and food. The animals were housed under standard conditions of a 12‐h light and 12‐h dark cycle, with a consistent temperature of 23°C ± 2°C.

After 1 week of acclimatization, the animals underwent treatment with CuSO_4_ (200 mg/kg body weight) in drinking water for 60 days. Moreover, a group of rats that received normal water was considered the control. After 60 days of treatment, the Cu administration was discontinued, and the Morris water maze (MWM) test was performed to evaluate the memory impairment in animals. Furthermore, six rats of the Cu‐treated group and six rats of the control group were sacrificed, and their blood, hippocampus, kidney, and liver tissues were harvested for further analysis. Subsequently, the remaining animals were randomly allocated into the following five groups, each consisting of six individuals (*n* = 6):

1. treated with 300 mg/kg body weight of *C. rotundus* extracts for 14 days through oral administration;
2. treated with 300 mg/kg body weight of *L. angustifolia* extract for 14 days through oral administration;
3. treated with vehicle (normal saline) for 14 days through oral administration (to investigate the effect of passing time in removing Cu from the body);
4. untreated group (termed CuSO_4_).

Following this 14‐day treatment period, the MWM test was employed to assess the impact of these extracts on memory performance restoration.

Noteworthy, the dose of 300 mg/kg for both extracts was chosen on the basis of previous in vivo studies demonstrating their neuroprotective and cognition‐enhancing effects (Mykhailenko et al. 2025; Sutalangka and Wattanathorn 2017). Moreover, all procedures conducted in this research were carried out in accordance with the guidelines of the ethics committee of the Neuroscience Research Center of Kerman University of Medical Sciences (IR.KMU.AEC.1403.026).

#### 2.4.1 MWM Test

This test was performed two times: first, after 60 days of exposure to CuSO_4_ to determine the induction of memory and learning impairments, and second, after 14 days of treatment with plant extracts. MWM involves a circular tank measuring 136 cm in diameter and 60 cm in height, situated in the center of a dimly lit testing room adorned with a few spatial signs. The water tank is currently filled with water and is being kept at a temperature of 25°C ± 2°C. The water level has been set to 20 cm below the rim of the tank. A stable circular platform, 10 cm in diameter, is positioned 1.5 cm below the water's surface in the water. In addition, a video camera, linked to an image analysis system, is positioned above the maze to track, digitize, and store data pertaining to the animals’ navigation within the maze for following behavioral analysis.

*Learning and Probe Phase of the MWM Task* During the learning trials, the rats were divided into four blocks, and each block had four trials. There was a 20‐min break between each block. A trial began by releasing the rat in one of the four randomly selected directions in the tank. The rats were given 1 min to swim and find the submerged platform. If a rat could not find the platform within a minute, it was gently guided to find it. The rat remained on the platform for 30 s between trials.

After 24 h of the last block, the retention phase (also known as the probe day) was carried out. In this phase, the platform was taken out of the pool, and the rats were given 1 min to search for it. This phase helps to determine whether the rats have memorized the location of the platform (Mohseni‐Moghaddam et al. 2022; Vorhees and Williams 2006).

### 2.5 The Measurement of Cu Accumulation

After the first 60 days of Cu exposure, as well as after 14 days of chelation therapy, to assess the accumulation of Cu in the animals’ tissues, the rats were anesthetized with ether vapors and immobilized through cervical dislocation. Subsequently, they were euthanized by exsanguination from the abdominal aorta, and their kidneys, brain, liver, spleen, and heart organs were harvested, weighed, and desiccated in an oven at 60°C for 3 days. To prepare the dried samples, 1.5 mL of HNO_3_ was added per 1 g of dry weight, followed by the addition of 1 mL of H_2_O_2_ for digestion. Next, the residues were diluted with deionized water to a volume of 100 mL. Finally, the Cu concentration in the organs was measured using the Varian atomic absorption spectrometers (F‐AAS and GF‐AAS).

### 2.6 Assessment of the MDA Levels

As MDA is one of the most common byproducts formed during oxidative stress–induced lipid peroxidation, we assessed the levels of thiobarbituric acid–reactive substances (TBARSs) to evaluate the oxidative stress in the animals’ tissues. The TBARS assay measures a range of reactive substances, including MDA, providing an overall indication of lipid peroxidation. For this purpose, each tissue sample weighing 0.1 g was homogenized in 0.1% trichloroacetic acid (TCA), followed by centrifugation of the homogenate at 15,000 rpm for 15 min. Subsequently, 4.0 mg of 0.5% thiobarbituric acid dissolved in 20% TCA was introduced to a 1 mg portion of the resulting supernatant. The mixture was then heated to 95°C and maintained at that temperature for 30 min, followed by cooling in an ice bath. The supernatant was subjected to centrifugation again at 10,000 rpm for 10 min. The absorbance of the resulting supernatant was recorded at 532 nm (Heath and Packer 1968). The results were normalized using absolute concentrations of MDA.

### 2.7 H2O2 Assay

The H_2_O_2_ levels, indicative of oxidative stress factors, were evaluated in the animals’ tissues using the method described by Velikova et al. (2000). In order to prepare the samples, we took 0.1 g of tissue and homogenized it using 1 mL of TCA with a pH level of 7.4. Following homogenization, the mixture was centrifuged at 10,000 × *g* for 10 min at 4°C. The H_2_O_2_ concentration in the tissue was determined by mixing 0.5 mL of tissue supernatant with an equal volume of 10 mM phosphate buffer at pH 7.4 in a cuvette. Subsequently, 1 mL of 1 mM potassium iodide was added, and the H_2_O_2_ concentration was measured spectrophotometrically at 390 nm. We used a standard curve of H_2_O_2_ to ensure accurate quantification.

### 2.8 Gene Expression Assay

#### 2.8.1 RNA Extraction and Reverse Transcription

The sacrifice of all animals took place 24 h after the last behavioral test. After removing the hippocampus, it was immediately frozen on dry ice and stored at a temperature of −80°C until the RNA extraction process took place. Total RNA was extracted from the tissue (five rats in each group) utilizing the Trizol reagent (Zaver Zist Azma, Iran). Furthermore, the concentration of the extracted RNA was evaluated using a NanoDrop spectrophotometer. Then, cDNA synthesis was conducted with Easy cDNA Synthesis Kit (ParsTous, Iran) according to the manufacturer's instructions.

#### 2.8.2 Quantitative Real‐Time Polymerase Chain Reaction (RT‐PCR)

RT‐PCR with the SYBR‐Green reporter dye was utilized to perform quantitative RT‐PCR. cDNA prepared from each sample was applied to quantify the mRNA expression genes using a Qiagen detection system (Qiagen, Germany). Glyceraldehyde 3‐phosphate dehydrogenase (GAPDH) was considered an internal control to standardize the expression levels of the target genes. The Real Q Plus 2× Master Mix (Solis BioDyne) was applied in the PCR reactions to amplify the cDNA sequences. Primers’ sequences, RT‐PCR product length, and their NCBI accession numbers are shown in Table 1. Besides, each sample was assessed in duplicate, and the average values were utilized in the following analysis. For evaluating the linearity and efficiency of PCR amplification, standard curves were created by increasing the cDNA amount. The relative mRNA levels were calculated on the basis of the 2^−ΔΔ^ *^CT^*.

**TABLE 1:** Primer sequences, real‐time polymerase chain reaction (RT‐PCR) fragment lengths, and NCBI accession numbers.

Gene name	Primer sequence	Size of PCR product	NCBI accession number
TNF‐α	F: ACCAGCAGATGGGCTGTACCTTAT R: ATGAAATGGCAAATCGGCTGACGG	107	NM_012675.3
IL‐1β	F: AAGACACGGGTTCCATGGTGAAGT R: TGGTACATCAGCACCTCTCAAGCA	97	NM_031512.2
BDNF	F: CGTGATCGAGGAGCTGTTGG R: CTGCTTCAGTTGGCCTTTCG	343	XM_008762078
IL‐6	F: CTGGTCTTCTGGAGTTCCGT R: TGGTCCTTAGCCACTCCTTCT	219	NM_012589.2
Caspase‐3	F: TGGTTCATCCAGTCACTTTG R: AATTCCGTGGCCACCTTCCG	101	XM_006253130.5
GAPDH	F: GTCTTCACCACCACGGAGAAGGC R: ATGCCAGTGAGCTTCCCGTTCAGC	392	NM_017008

### 2.9 Assessment of the Serum Levels of Biochemical Factors

For the assessment of blood biochemical factors, the animals were anesthetized, and blood samples were obtained via cardiac puncture, followed by centrifugation to isolate the serum. Subsequently, various parameters, including creatinine, urea, alanine aminotransferase (ALT), and aspartate aminotransferase (AST), were assessed using standard kits (KONELAB 20XT, Finland), following the manufacturer's instructions.

### 2.10 Statistical Analysis

For the purpose of analyzing the data, the statistical software SPSS 19.0 was used. The results were presented in the form of mean ± SEM. To determine the statistical differences among different groups, the one‐way analysis of variance (ANOVA) was conducted, followed by the Tukey post hoc test.

## 3 Results

### 3.1 Antioxidant Activity of Herbal Extracts

**FIGURE 1:**
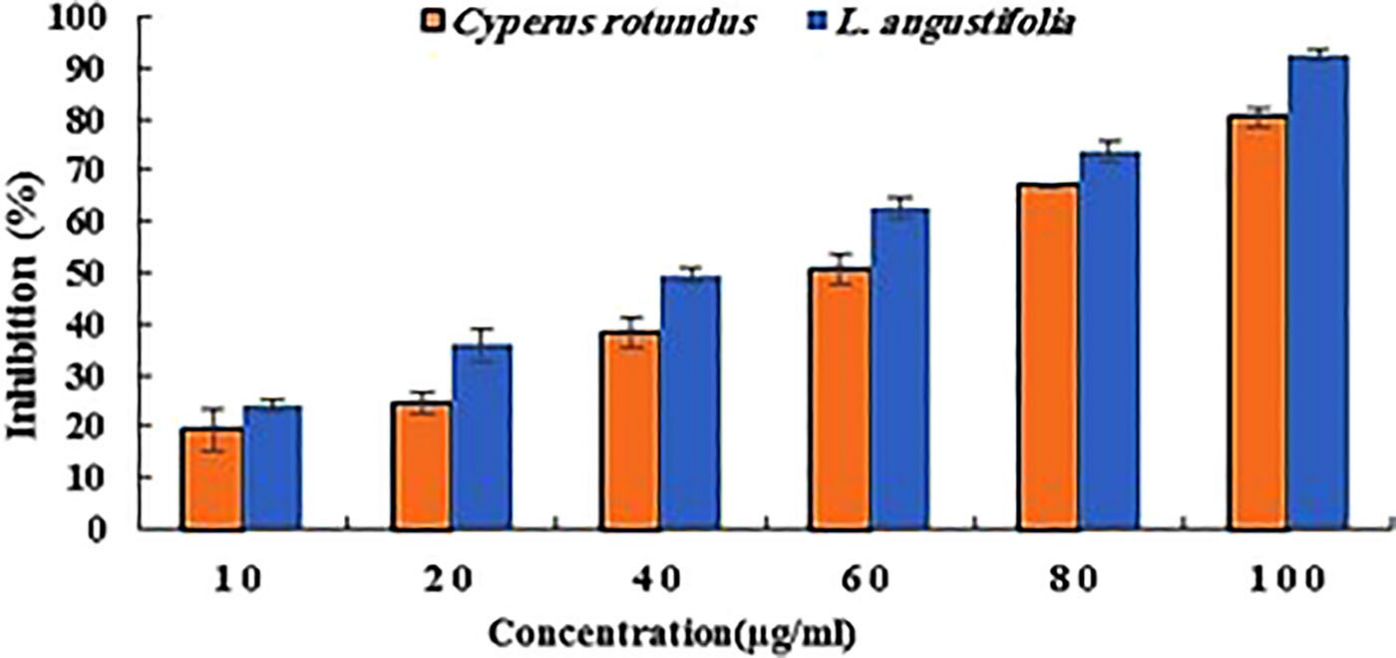
DPPH free radical scavenging activity of *Cyperus rotundus* and *Lavandula angustifolia* extracts and different concentrations.

The results of the DPPH radical scavenging assay of both extracts are shown in Figure 1. According to the results, these extracts showed considerable activity in inhibiting the radicals dose‐dependently. The concentration of 100 µg/mL showed the best antioxidant activity regarding both extracts. In this concentration, the extracts of *C. rotundus* and *L. angustifolia* inhibited 80% and 92% of DPPH radicals, respectively. Furthermore, the calculation of the IC_50_ value revealed that the *L. angustifolia* extract with an IC_50_ of 42 µg/mL had better antioxidant activity than *C. rotundus* with an IC_50_ of 56 µg/mL.

### 3.2 Learning and Memory Functions (MWM)

To determine the effects of CuSO_4_ on the animals’ memory performance, the MWM test was performed. According to Table 2, the rats exposed to CuSO_4_ exhibited significant learning deficiency, as evidenced by increased distance and duration of travel to locate the hidden platform during the training phase, compared to the control group. Moreover, the presence of CuSO_4_ in the environment caused the rats to exhibit a lower likelihood of entering the target quadrant, travel a lesser distance there, and spend less time in the area as compared to the control group during the probe test. This points toward the occurrence of a memory deficiency. However, continued treatment of the animals with the plant extracts for an additional 14 days demonstrated that both *L. angustifolia* and *C. rotundus* extracts were effective in significantly reducing memory and learning impairment symptoms in rats.

**TABLE 2:** Effects of 60‐day exposure to CuSO_4_ and a further 14‐day treatment with *Lavandula angustifolia* and *Cyperus rotundus* extracts and DFX on spatial learning and memory performance of rats.

	Treatments			Performance		
		Learning		Memory		
		Duration (s)	Distance (m)	Number of entries	Times (s)	Distance (m)
60‐day	Control	4.7 ± 0.13	0.94 ± 0.19	8 ± 0.57	26.1 ± 1.8	3.4 ± 0.3
	CuSO_4_	16.8 ± 1.16^***^	2.11 ± 0.2^**^	4.4 ± 0.36^***^	18.4 ± 1.9^***^	2.4 ± 0.2^**^
Further 14‐day	Vehicle (Control)	16.2 ± 1.15^***^	2.12 ± 0.21^**^	4.6 ± 0.48^***^	18.6 ± 1.6^***^	2.6 ± 0.3^*^
	*L. angustifolia*	12.4 ± 1.52^** #^	1.56 ± 0.22^* #^	6.1 ± 0.59^* ##^	23.7 ± 1.8^* ##^	4 ± 0.2^#^
	*C. rotundus*	14.1 ± 1.02^** #^	1.64 ± 0.28^* #^	6 ± 0.39^* ##^	21.3 ± 2.13^* #^	4.08 ± 0.3^#^

*Note*: Values are expressed as mean ± SEM. Statistical significance is indicated as follows: *p < 0.05, **p < 0.01, and *** *p* < 0.001 when compared to the control group. Furthermore, #*p* < 0.05, ##*p* < 0.01, and ###*p* < 0.001 when compared to both the CuSO_4_ and vehicle groups.

### 3.3 The Cu Accumulation

The distribution of Cu in animals’ organs, before and after chelation therapies, is shown in Table 3. On the basis of the results, the Cu content in tissues was meaningfully increased in the animals exposed to CuSO_4_. This increase in the kidneys (2.25 mg/kg), liver (0.66 mg/kg), and brain (0.5 mg/kg) was dominant when compared to the corresponding control. Among these, by far the largest amount of Cu was accumulated in the kidneys, with 5.3 mg/kg. However, after 14 days of chelation therapy with *L. angustifolia* and *C. rotundus* extracts, the Cu level in this organ reduced to 3.27, 2.83, and 2.9 mg/kg, respectively, indicating a similar effect for all treatments. A considerable reduction was also observed regarding other organs of treated rats compared to the control (vehicle).

**TABLE 3:** The copper distribution in animals’ organs before and after chelation therapies.

		Cu level (mg/kg)				
	Groups	Heart	Kidneys	Brain	Spleen	Liver
60 days	Control	1.071 ± 0.03	2.36 ± 0.1	0.75 ± 0.02	0.47 ± 0.05	1.19 ± 0.07
	CuSO_4_	1.33 ± 0.11^*^	5.3 ± 0.13^***^	1.24 ± 0.03^***^	0.67 ± 0.02^**^	1.85 ± 0.09^**^
Further 14‐day	Vehicle	1.37 ± 0.05^*^	4.56 ± 0.13^***^	1.18 ± 0.1^***^	0.69 ± 0.02^**^	1.76 ± 0.09^**^
	*L. angustifolia*	1.21 ± 0.03	3.27 ± 0.3^##^	0.94 ± 0.02^##^	0.56 ± 0.01	1.45 ± 0.03^*^
	*C. rotundus*	1.02 ± 0.02^#^	2.83 ± 0.3^###^	1.0 ± 0.03^* #^	0.66 ± 0.08	1.25 ± 0.08^##^

*Note*: Values are shown as mean ± SEM. Statistical significance is denoted as follows: *p < 0.05, **p < 0.01, and ***p < 0.001 when compared to the control group. Additionally, #*p* < 0.05, ##*p* < 0.01, and ###*p* < 0.001 compared to both the CuSO_4_ and vehicle groups.

### 3.4 MDA and H_2_O_2_ Levels

**FIGURE 2:**
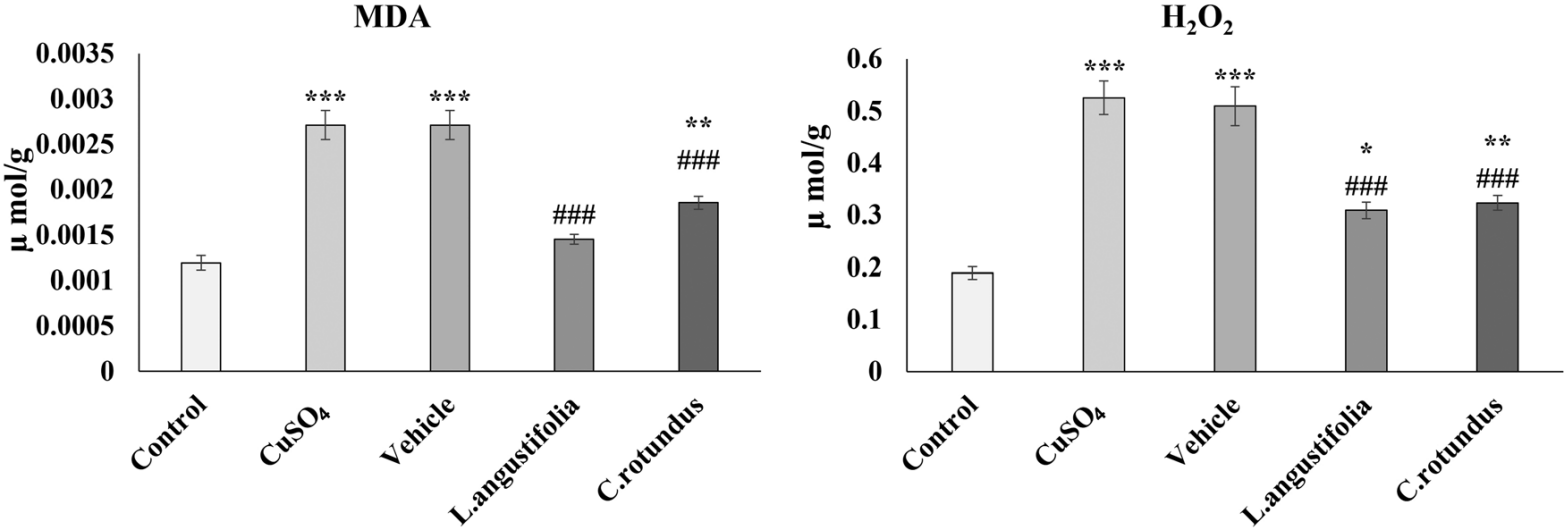
Impact of CuSO_4_ and herbal extracts on MDA and H_2_O_2_ levels in the rat hippocampus. Values are shown as mean ± SEM. Statistical significance levels are denoted as follows: *p < 0.05, **p < 0.01, and ***p < 0.001 when compared to the control group. Furthermore, #*p* < 0.05, ##*p* < 0.01, and ###*p* < 0.001 in comparison to both the CuSO_4_ and vehicle groups. MDA, malondialdehyde.

Along with H_2_O_2_, we evaluated oxidative stress in animal tissues by measuring TBARS, which indicate lipid peroxidation, including levels of MDA. Contents of MDA and H_2_O_2_ in the hippocampus of rats were measured to assess the severity of oxidative stress in the different experimental groups (Figure 2). As results show, MDA and H_2_O_2_ levels significantly increased by 2‐ to 3‐fold in the animals’ hippocampus after 60 days of exposure to CuSO_4_ compared to the control group (*p* < 0.001). However, treating the animals with the plant extracts considerably decreased these features, so after 14 days, the MDA and H_2_O_2_ levels in the treated animals were approximately at the same levels as the control group.

### 3.5 Gene Expression

**FIGURE 3:**
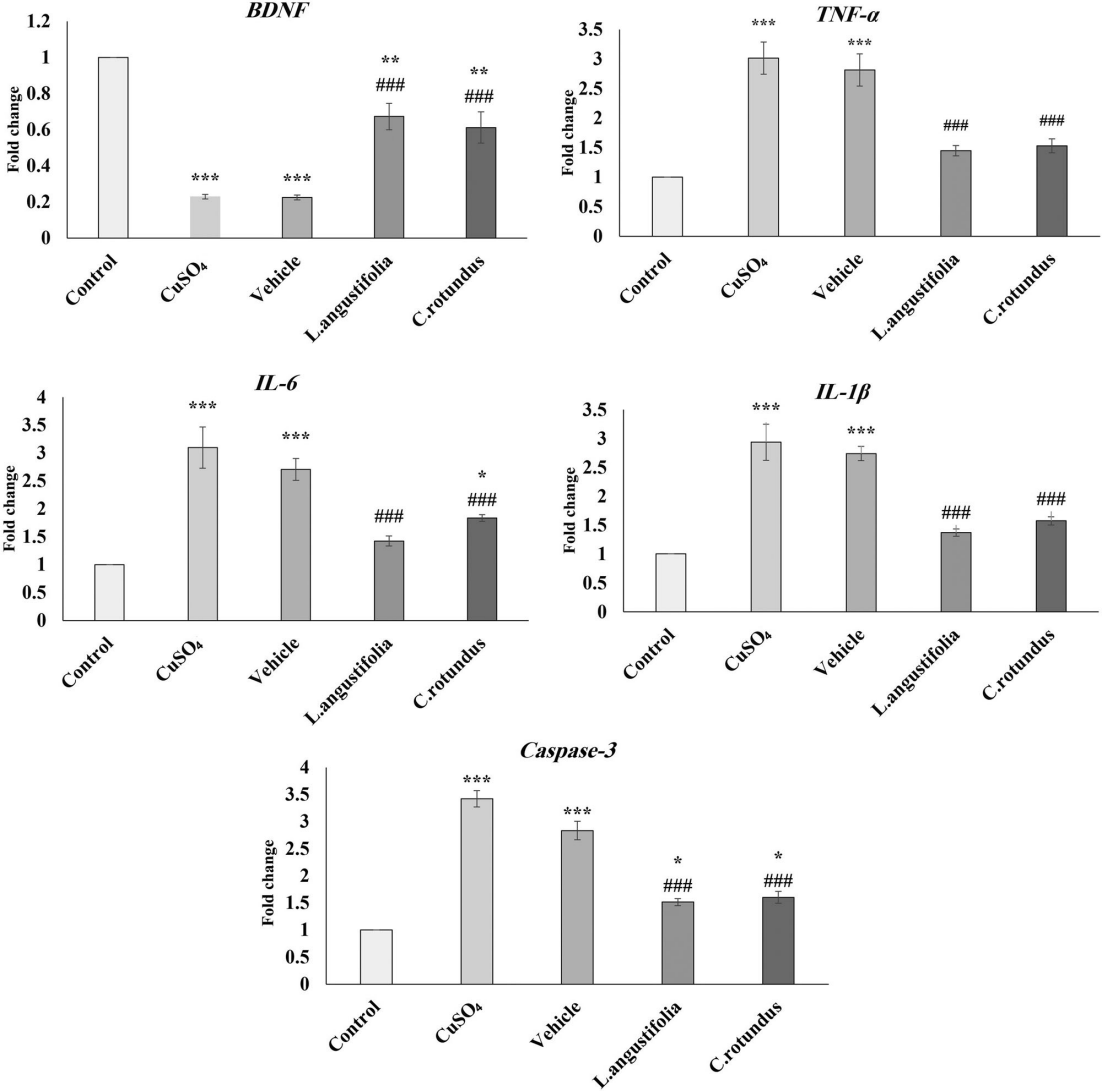
Effects of CuSO_4_ and treatment with *Lavandula angustifolia* and *Cyperus rotundus* extracts on the gene expression of BDNF, TNF‐α, IL‐6, IL‐1β, and Caspase‐3 in the rats’ hippocamp. Values are shown as mean ± SEM. Statistical significance levels are denoted as follows: *p < 0.05, **p < 0.01, and ***p < 0.001 in comparison with the control (vehicle) group. In addition, ###*p* < 0.001 when compared to CuSO_4_ and vehicle groups.

As shown in Figure 3, the BDNF expression in the hippocampus of animals subjected to CuSO_4_ decreased, whereas the expression of IL‐6, IL‐1β, TNF‐α, and Caspase‐3 significantly increased in comparison to the control group. However, the subsequent 14 days of treatment with the *L. angustifolia* and *C. rotundus* extracts could significantly attenuate this effect. For example, both extracts could increase the BDNF levels in the treated groups by about 20% compared to the CuSO_4_ group. The obtained results also showed that the overexpression of TNF‐α, IL‐6, IL‐1β, and Caspase‐3 was significantly downregulated following treatment (on average 50%) compared to the control (vehicle) group.

### 3.6 Serum Levels of Biochemical Parameters

The results presented in Table 4 indicate that exposure to CuSO_4_ significantly increased the levels of AST, ALT, and urea in animal serum by approximately 100% compared to the control group. On the other hand, treating the animals with *L. angustifolia* and *C. rotundus* extracts for 14 consecutive days significantly decreased serum levels of these factors compared to the group. Noteworthy, neither exposure to CuSO_4_ nor treatment with plant extracts significantly affected creatinine levels.

**TABLE 4:** Effect of the extract on serum biochemical factors.

Treatments		Biochemical factors			
		ALT	AST	Urea	Creatinine
**60 days**	Control	63.3 ± 2.9	198 ± 1.7	28.3 ± 1.4	0.13 ± 0.01
	CuSO_4_	125.3 ± 9.2^***^	373 ± 14.7^***^	57 ± 1.5^***^	0.13 ± 0.03
**Further 14‐day**	Vehicle	114.3 ± 3.3^***^	315 ± 10.5^***^	63.6 ± 2.4^***^	0.1 ± 0
	*L. angustifolia*	86 ± 0.5^##^	239.3 ± 7.7^##^	37.3 ± 1.4^###^	0.1 ± 0
	*C. rotundus*	80 ± 2.3^###^	205 ± 19.7^###^	33.6 ± 2.3^###^	0.13 ± 0.04

*Note*: Values are shown as mean ± SEM. Statistical significance levels are denoted as follows: ^***^ *p* < 0.001 in comparison with the control group. In addition, ^##^ *p* < 0.01 and ^###^ *p* < 0.001 when compared to the CuSO_4_ and vehicle group.

## 4 Discussion

The presence of heavy metals in wastewater is a common issue that poses a significant threat to the environment and human health. Contaminated wastewater can leach heavy metals into groundwater or irrigation systems, leading to bioaccumulation in food and drinking water sources. Among the heavy metals frequently detected in wastewater, arsenic, cadmium, chromium, copper, lead, nickel, and zinc are the most notable. Accumulation of these elements can have adverse impacts on both human health and the environment (Jaishankar et al. 2014). In particular, the tissue accumulation of Cu causes perturbations in metal homeostasis, resulting in an array of cellular disturbances, increased free radical production, and oxidative stress (Driessnack et al. 2017). These findings emphasize the importance of searching for effective chelating and antioxidant medications with minimal side effects to counteract the detrimental effects of Cu. This study aimed to evaluate the antioxidant properties of extracts from *L. angustifolia* and *C. rotundus*, as well as their ability to mitigate memory and learning impairments induced by Cu.

The findings of the study suggest a comprehensive relationship between Cu exposure, oxidative stress, cognitive impairment, and organ damage. As demonstrated by atomic absorption spectrometry, prolonged exposure to CuSO_4_ resulted in increased Cu levels in the brain, leading to oxidative stress and subsequent impairments in learning and memory. The observed elevation in MDA and H_2_O_2_ levels in the hippocampus further supports the role of oxidative stress in these cognitive impairments (Khorrami et al. 2023). It is well known that the dysregulation of Cu_2_^+^ homeostasis is widely recognized as a significant factor in the onset of neurological disorders (Travaglia et al. 2012). A preclinical study showed that even small amounts of Cu in drinking water could lead to amyloid‐β (Aβ) accumulation and significantly delay learning ability (Sensi et al. 2018). Moreover, a recent study by Pan et al. (2022) showed that prolonged exposure to Cu could lead to mitochondria‐mediated apoptosis in the hearts of mice. In cases of acute poisoning, Cu binds to albumin and is absorbed by the liver, kidneys, brain, and red blood cells (RBCs). The clinical progression in such individuals is marked by challenges, such as intravascular hemolysis, jaundice, renal failure, methemoglobinemia, and symptoms affecting the central nervous system (CNS) (Malik and Mansur 2011).

Additionally, on the basis of the results, hepatic and kidney enzymes, including AST, ALT, and urea, as quantitative markers of liver and kidney injury, increased following CuSO_4_ exposure compared to the control group. This can be related to the accumulation of Cu in these organs, indicating potential liver and kidney injury and highlighting the systemic impact of Cu accumulation. According to previous studies, animals exposed to Cu show high levels of ALT, AST, urea, and creatinine, indicating liver and kidney function disturbance (Khushboo et al. 2018). Moreover, consistent with our results, a recent study has presented evidence suggesting that an excess of Cu can initiate autophagy and apoptosis through the mitochondrial pathway by inducing oxidative stress in the kidneys of rats (Wan et al. 2020).

Our research findings indicate a decrease in mRNA expression of key antioxidant factors, such as BDNF, in the hippocampal tissue, along with an increase in inflammatory mediators like IL‐6, IL1‐B, and TNF‐α in the tissue of animals exposed to Cu. We also discovered an upregulation of the Caspase‐3 gene, which is an apoptotic protein. These observations suggest that neuroinflammation and apoptotic pathways are involved in the animals’ brains, which could explain the cognitive deficits we observed. It has been revealed that Cu accumulation can induce oxidative stress by generating ROS, which in turn can activate pro‐inflammatory pathways, such as NF‐κB, MAPKs, and NLRP3 inflammasome. This cascade can lead to the upregulation of pro‐inflammatory mediators like TNF‐α, IL‐6, and IL‐1β1 (Deng et al. 2023).

Additionally, there is solid evidence that the rate of cognitive decline in AD patients is specifically associated with decreased serum BDNF levels (Gao et al. 2022). Moreover, it is likely that exposure to Cu can affect the brain's inflammatory responses and hinder the removal of Aβ, which is associated with cognitive impairments. A study conducted by Kitazawa et al. (2016) demonstrated that exposure of murine monocyte BV2 cells to Cu led to reduced phagocytic activation in response to fibrillar Aβ or lipopolysaccharide. Simultaneously, there was a notable increase in the secretion of pro‐inflammatory cytokines, such as IL‐1β, TNF‐α, and IL‐6. Aligning with their in vitro findings, in a mouse model, Cu exposure resulted in a significant rise in neuroinflammation and a downregulation of Low‐density lipoprotein receptor‐related protein 1 (LRP1) in the brain (Kitazawa et al. 2016). The downregulation of LRP1 in the brain, resulting in impaired clearance of Aβ peptides, can contribute to the pathological processes associated with AD, including the accumulation of amyloid plaques, neurotoxicity, neuroinflammation, and cognitive decline (Liu et al. 2017; Samandari‐Bahraseman et al. 2019).

On the other hand, despite Cu's detrimental effects, we found that 14 days of treatment with *L. angustifolia* and *C. rotundus* extracts could reduce Cu accumulation and ameliorate learning and memory impairments. Furthermore, after the treatment period, the Cu content accumulated in the kidney, liver, and brain of rats was markedly reduced in comparison with the non‐treated groups. These findings are consistent with previous studies, indicating the effectiveness of plant extracts in reducing the detrimental effects of heavy metal toxicity (Udo et al. 2023). The effects of *L. angustifolia* and *C. rotundus* plant extracts can be attributed to their chelating and antioxidant activity. As we found, these extracts were able to scavenge more than 80% of DPPH radicals. However, the IC_50_ value of the *L. angustifolia* extract was lower, indicating its higher antioxidant capacity (Khorrami et al. 2019). Our findings are in good agreement with some similar previous investigations, demonstrating that these plant extracts have free radical scavenging activity (Bordoloi and Basumatary 2016; Tousson and El‐Gharbawy 2023). Research has also shown that lavender oil has protective properties against renal ischemia–reperfusion injury by targeting both pro‐ and anti‐inflammatory cytokines. It also aids in the reduction of oxidative stress factors and apoptosis (Aboutaleb et al. 2019). Moreover, it has been recently shown that spatial memory increased in AD rats treated with *C. rotundus* extract (Shakerin et al. 2020). Generally, the administration of plant extracts with antioxidant activity has been shown to inhibit Caspase‐3 activation and the apoptosis process in AD mouse models (S. K. Singh et al. 2017).

The precise mechanism of action through which the extracts function has not been fully understood. However, these extracts are notable for their variety of phytochemicals, which significantly contribute to their antioxidant, anti‐inflammatory, and neuroprotective properties. *L. angustifolia* extract is primarily composed of monoterpenes, with linalool, linalyl acetate, borneol, and alpha‐terpineol being the main constituents. Additionally, it contains phenolic acids, including coumaric acid, glycolic acid, valeric acid, and particularly rosmarinic acid, which some studies have found to be present at levels of up to 10 mg/g in the extract (Batiha et al. 2023; Habán et al. 2023). Similarly, *C. rotundus* extract showcases a rich phytochemical profile that includes sesquiterpenes, flavonoids, and phenolic acids. Detailed phytochemical analyses have identified compounds such as α‐cyperone and cyperene as prominent sesquiterpenes, whereas flavonoids like quercetin and phenolic acids such as gallic acid and *p*‐coumaric acid have also been reported. Quantitative studies indicate that the concentrations of these bioactive compounds can exceed 50 mg/g in certain cases (Babiaka et al. 2023, Babiaka et al. 2025). These compounds exert their bioactivity primarily through scavenging free radicals, reducing lipid peroxidation, and modulating inflammatory pathways, which collectively contribute to the mitigation of oxidative stress and, consequently, copper‐induced neurodegeneration.

Our findings suggest that the observed cognitive improvement in Cu‐exposed rats treated with plant extracts is likely due to the reduction of neuroinflammation and functional recovery of “damaged but surviving” neurons, rather than regeneration of lost neurons. This is supported by studies indicating that neuroinflammation contributes to cognitive decline and that reducing inflammation can improve cognitive function (Bollen et al. 2017). Additionally, Cu's role as a neuromodulator, particularly its ability to bind to dopamine receptors and potentially disrupt reward and learning circuits, is well documented (D'Ambrosi and Rossi 2015; Tapiero et al. 2003). This disruption could be relevant to the cognitive deficits observed in our study, further emphasizing the importance of mitigating Cu toxicity to preserve neuronal function.

On the basis of our findings, the plant extracts were able to significantly mitigate the adverse effects of long‐term Cu exposure in rats. Plant extracts with chelating agents offer several advantages over common chemical chelating agents. They are sourced from natural materials, reducing environmental impact, and typically have fewer side effects (Khorrami et al. 2018, Khorrami et al. 2020). Additionally, due to their complex composition, which includes a variety of bioactive compounds that can work synergistically, they can provide a broader and potentially more effective chelation profile. These compounds can modulate multiple biological pathways, reducing the likelihood of adverse effects. Additionally, the antioxidant and anti‐inflammatory properties of many plant extracts can counteract the potential side effects associated with chelation therapy (Mehrandish et al. 2019). However, more research is needed to precisely understand the benefits and limitations of plant extracts with chelating agents.

## 5 Conclusion

The exposure to high levels of Cu leads to spatial learning and memory disorders, as well as liver and kidney dysfunctions. An excessive amount of CuSO_4_ raises the H_2_O_2_ and MDA levels. It also declines the expression of the BDNF gene while increasing the expression of IL‐6, IL1‐B, Caspase‐3, and TNF‐α. According to the research findings, the *L. angustifolia* and *C. rotundus* extracts have the potential to remove Cu from the body and reduce its harmful effects. These extracts increase the expression of the BDNF gene while decreasing the expression of IL‐6, IL1‐B, Caspase‐3, and TNF‐α genes in the hippocampus. Additionally, they reduce MDA and H_2_O_2_ levels in the rat hippocampus. The extracts offer a cost‐effective and natural reservoir of antioxidants and chelating agents that can be utilized for both food and pharmaceutical applications.

## Author Contributions

**Mandana Salari**: investigation, writing – original draft, methodology, resources. **Faezeh Dahoee**: investigation, validation. **Seyed Jamilaldin Fatemi**: conceptualization, investigation, writing – review and editing, supervision, resources. **Manijeh Dogani**: methodology, formal analysis.

## Conflicts of Interest

The authors declare no conflicts of interest.

## Peer Review

The peer review history for this article is available at: https://publons.com/publon/10.1002/brb3.70624.

## Data Availability

Data sharing is not applicable to this article as no new data were created or analyzed in this study.
